# Effects of predation risk on the sensory asymmetries and defensive strategies of *Bufotes balearicus* tadpoles

**DOI:** 10.1007/s10071-022-01687-5

**Published:** 2022-09-13

**Authors:** Andrea Gazzola, Bianca Guadin, Alessandro Balestrieri, Daniele Pellitteri-Rosa

**Affiliations:** Department of Earth and Environmental Sciences, University of Pavia, Pavia, Italy

**Keywords:** Alien species, Amphibians, Anti-predatory behaviour, Lateralization, Rotational preference, Tadpoles

## Abstract

Lateralization consists of the differential use of bilateral organs or limbs and is well described in many taxa and in several contexts. Common ecological frameworks where it can be observed are foraging and predatory ones, with benefits related to both visual and auditory lateralization such as faster response or increasing neural processing ability. Anuran amphibians are considered relevant models for investigating lateralization, due to their great ecological variety and the possibility of easily being raised under laboratory conditions. By adopting the “rotational preference test”, we used Balearic green toad tadpoles to test the effects of behavioural defensive responses triggered by different predator types (native vs alien, i.e. dragonfly larvae *Aeshna cyanea* and adult red swamp crayfish *Procambarus clarkii*) and diets (fasted vs. tadpole-fed predators) on their lateralization. We recorded tadpoles’ responses to five different chemical cues: clean water (control treatment), fasted dragonfly larvae and crayfish, and tadpole-fed dragonfly larvae and crayfish. Green toad tadpoles did not show a bias in a predominant direction, although lateralization occurred at the individual level, as shown by the intensity index (*L*_A_). Perceived predation risk was the highest in tadpoles exposed to the combined chemical cues of conspecific prey and native predators, which elicited both changes in the intensity of lateralization and a marked reduction in tadpoles’ activity level. Our results suggest that contextual predation threat may induce very rapid changes in the expression of asymmetries at the individual level, and might play a role as part of the complex defensive strategies adopted by prey in the attempt to escape predators.

## Introduction

Lateralization refers to the specialization of the two sides of the brain to carry out different neural, physiological and behavioural activities. As each side of the nervous system controls the contralateral part of the body (i.e., the left side controls the right half of the body and vice versa), lateralization can be expressed as motor asymmetries or limb use preferences (review in Rogers et al. 2013). In most vertebrates, the right side of the brain is responsible for impulsive and direct responses, while the left side is the one that typically controls functions requiring some sort of processing or elaboration of different stimuli (MacNeilage et al. 2009). When necessary, the left hemisphere can inhibit the activity of its counterpart (Rogers 2002; Vallortigara and Rogers 2020).

Although the occurrence, extent and effects of lateralization have been investigated in a wide range of animal taxa (e.g.: several brain functions in rats, Denenberg 1983; vision and feeding in pigeons and domestic chicks, Güntürkün and Kesch 1987; Diekamp et al. 2005; Vallortigara et al. 2001; aggressive responses in the lizard genus *Anolis*, Deckel 1995; escape behaviour in the teleost fish *Girardinus falcatus*, Cantalupo et al. 1995; righting behaviour in *Testudo hermanni* and *Emys orbicularis*, Stancher et al. 2006, Pellitteri-Rosa and Gazzola 2018; monitoring of predators by *Podarcis muralis*, Bonati et al. 2010), it is not always obvious what are the pros and cons of such behavioural asymmetries. For example, sharp side biases could affect foraging: an animal which preferentially uses the left eye to scan for food may be expected to be poorly reactive to potential prey running past its right eye, a condition which, in the long term, may lead to starvation (Vallortigara and Rogers 2005; Vallortigara and Versace 2017).

From a prey’s point of view, potential costs of lateralization appear to be even higher: predation risk varies greatly in both space and time (Lima and Bednekoff 1999), and, moreover, predators may learn to attack on their prey’s defective side (Corballis 1998). Hence, sensory asymmetries may be expected to undergo negative selection (Vallortigara and Rogers 2005; Dadda et al. 2010). Notwithstanding, since lateralization is widespread in many animal groups, including extinct species (Babcock 1993; Reisz et al. 2020), we can safely assume that evolutionary pros counterbalanced or outweighed cons (Rogers 2000). Highly lateralized brains may allow to manage multiple simultaneous neural activities, enhancing the overall cognitive performance (Levy 1977; Rogers 2002; Rogers et al. 2004; Dadda and Bisazza 2006; Magat and Brown 2009), and preventing incompatible brain responses from working at cross purposes (Vallortigara 2000; Vallortigara and Rogers 2005; Stancher et al. 2018).

Several species show a lateral preference when detecting a predator or escaping attacks (e.g.: Yamashita et al. 2000; Lippolis et al. 2002; Martín et al. 2010), and lateralization tends to increase with predation risk (Brown et al. 2004; Ferrari et al. 2017), enhancing prey survival (Ferrari et al. 2015a). Coupled with frequency-dependent predation costs, enhanced coordination with conspecifics may have led to the unbalanced proportion of left- and right-lateralized individuals usually observed in social species (Ghirlanda and Vallortigara 2004; Ghirlanda et al. 2009; Frasnelli and Vallortigara 2018).

Since the 1990’s, anuran amphibians have been considered excellent models for investigating lateralization, due to their great ecological diversity and the possibility of easily raising them under laboratory conditions. Previous studies have analysed preferences for limb use (Bisazza et al. 1996, 1997; Sovrano 2007), predator avoidance (Lippolis et al. 2002), emetic behaviour (Naitoh and Wassersug 1996), vocalization (Bauer 1993), righting (Bisazza et al. 1996, 1997; Robins et al. 1998) and other motor responses (reviewed by Malashichev and Robins 2018). Vallortigara et al. (1998) recorded that *Bufo bufo* and *Bufotes viridis* toads show a clear right-bias in their anti-predatory behaviour, while agonistic behaviour is mainly induced by conspecifics entering their left hemifield (see also Robins et al. 1998).

While adult anurans are mostly solitary, except for the mating season, their larvae often live in large groups, suggesting that lateralization may be enhanced by the need for coordinated movements. Wassersug et al. (1999) analysed turning biases in the escape responses of *Rana catesbeiana* and *Xenopus laevis* tadpoles and recorded some asymmetries in both the morphology of the former species’ branchial chambers and motor responses. Notwithstanding, the hypothesis that the left bias depended on morphological asymmetries was falsified when Yamashita et al. (2000) demonstrated that tadpoles of *Microhyla ornate* are lateralized in their turning behaviour despite being externally symmetrical. This result suggested that behavioural lateralization may be linked to phylogeny (*R. catesbeiana* and *M. ornata* showing a closer evolutionary relation than *X. laevis* and *M. ornata*) rather than morphology, even though body asymmetry and laterality may have been connected at some point during the latter trait’s first evolutionary steps (Goree and Wassersug 2001).

Supporting the role played by social factors (Ghirlanda and Vallortigara 2004), turning bias seems to be expressed differently in the various stages of development (Oseen et al. 2001), and progressively recedes as tadpoles reach metamorphosis, probably because the appearance of limbs is accompanied by the alteration and reconnection of neural networks (Malashichev and Wassersug 2004; Malashichev and Robins 2018). Lucon-Xiccato et al. (2017) demonstrated that *Lithobates sylvaticus* tadpoles reared in a high predation risk environment show intense laterality in their swimming behaviour compared to tadpoles maintained under low risk, suggesting that developmental plasticity may enhance an individual’s chance to escape predation.

Using Balearic green toad *Bufotes balearicus* as a model species and the “rotational preference test”, which has been used in several taxa, including anuran larvae (Sobel et al. 1994; Bisazza and Vallortigara 1997; Blackiston and Levin 2013; Lucon-Xiccato et al. 2017; Bolis et al. 2020; Gazzola et al. 2021), we aimed to test the effects of behavioural defensive responses elicited by different predator types (native vs alien) and diets (fasted vs. tadpole-fed predators) on tadpole lateralization. Based on previous studies, we expected tadpoles’ level of activity to be strongly influenced by tadpole-fed, native predators and predicted lateralization to increase consequent to exposure to cues matching an actual risk of predation.

## Methods

### Sample collection

In May 2020, 10 freshly laid green toad strings were collected from a network of canals flowing in an intensively cultivated area south of Milan (45° 26′ N, 9° 20′ E, Lombardy region, Northern Italy). In the laboratory, each clutch was kept in 15 l tubs filled with 10 l of dechlorinated water and, after hatching, tadpoles were transferred into three containers (150 l), filled with 80 l of dechlorinated water. Throughout the rearing period, tadpoles were provided with food ad libitum, consisting of dry grass pellets (rabbit food).

Eight late instar dragonfly larvae (*Aeshna cyanea*) and eight adult red swamp crayfish (*Procambarus clarkii*) were collected from artificial ponds located inside the protected natural area “Bosco del Vignolo” (45° 13′ N, 8° 56′ E), using dip-nets. In the laboratory, dragonfly larvae were kept individually in 0.8 l plastic tubs filled with 0.5 of aged tap water, while each crayfish was kept in an 11 l plastic tub filled with 2 l of aged tap water.

The permits to perform this study were obtained from the Italian Ministry of Environment, Land and Sea (0006075–23/03/2018—PNM).

### Experimental design

We recorded tadpoles’ behavioural responses to five different olfactory cues: clean water (control treatment), fasted dragonfly larvae, tadpole-fed dragonfly larvae, fasted crayfish and tadpole-fed crayfish. Before the onset of the experiment, tadpoles were visually selected to form groups at the same developmental stage (Gosner’s stage 26–28) and size (visually estimated), which were then moved to a 150 l container filled with 100 l of aged water (*n* = 200). Each trial consisted of a grid composed of 10 opaque, cylindrical experimental cups (12 cm inside diameter) filled with 200 ml of aged tap water, which were positioned inside a large white opaque plastic container (63 × 85 × 50 cm) to avoid disturbance from external sources. A video camera (digital Canon Legria) was positioned 1.5 m above the grid level. The plastic container was illuminated uniformly on all sides. Treatments (two replicates per trial) were randomly distributed within each grid. To assess the activity of the larvae before and after the infusion of cues, each trial lasted 40 min in total. Tadpoles were individually positioned into the cups and left to acclimatize for 20 min. Then they were recorded for 20 min both before (pre-stimulus) and after (post-stimulus) the infusion of the cue (2 ml), which was gently injected by a syringe. Each tadpole was tested once, for a total of 125 tadpoles (25 for each chemical stimulus).

### Preparation of olfactory cues

To assess predation risk, anuran larvae generally rely on water-borne chemical cues (Kats and Dill 1998), consisting of predator-specific odours, cues released by conspecifics, or, more frequently, a combination of both (Fraker et al. 2009; Hettyey et al. 2015). Several studies have shown that fed predators commonly elicit stronger antipredator defences than starved predators (Petranka and Hayes 1998; Van Buskirk and Arioli 2002; Schoeppner and Relyea 2009; Gazzola et al. 2018a). As a predator may become chemically ‘labelled’ by its diet via learning processes, recognition of a novel predator can be facilitated by the association of its specific cues to those released by conspecifics (reviewed in Ferrari et al. 2010; Mitchell et al. 2017).

To obtain the odour-stimuli, four specimens of each predator were assigned randomly to each of the two diet treatments. Fed-predators were provided for three consecutive days, at 6:00 pm, with two green toad tadpoles (total wet weight about 150 mg) each. The day after, one hour before the onset of the experiment, an aliquot of water (50 ml) was collected from each predator tub and aliquots from the same treatment were poured into a new container. The resulting mixtures were used as odour stimulus during behavioural trials (2 ml per cup). Every time, after the collection of the chemical stimulus, the water of predator tubs was renewed.

### Data collection and statistical analysis

To assess behavioural lateralization, all recordings were visually inspected by the same observer, who was blind to the chemical treatment provided to each experimental unit. A circle (6 cm in diameter) was overlapped, in the videos, on the centre of each cup and the time spent by each tadpole swimming inside the circular crown, in either clockwise or counterclockwise direction, was recorded during both the pre- and post-stimulus periods (Blackiston and Levin 2013; Bolis et al. 2020). Rotational preference was then estimated through lateralization directionality and intensity (Lucon-Xiccato et al. 2017; Bolis et al. 2020). The first parameter (*L*_R_ index), which refers to directionality, was calculated with the formula: (clockwise swimming time − counterclockwise swimming time)/(clockwise swimming time + counterclockwise swimming time) × 100. When *L*_R_ is close to zero the tested individual does not show any rotational preference. The intensity of lateralization (*L*_A_ index) was obtained by taking the modulus of *L*_R_ (*L*_A_ =|*L*_R_|). Both indexes were calculated before and after adding the stimulus. While *L*_R_ refers to population-level lateralization, *L*_A_ allows to compare the amount of lateralization (regardless of its direction) among groups at the individual level.

To investigate locomotor responses (i.e., variation in activity level) all video clips were analysed by a source executable software for image-based tracking (ToxTrac; Rodriquez et al. 2017), which provides locomotor information by recording the *x* and *y* coordinates of the central point of each tadpole every 0.04 s.

The variation in the activity level of tadpoles was assessed by two variables provided by the tracking software: total time frozen and average acceleration. The first variable was recorded to highlight the time spent inactive (motionless) by tadpoles as a behavioural response that facilitates the avoidance of detection by the predator, while the average acceleration was intended to provide an estimate of the intensity with which tadpoles changed their state, from motionless to active.

Statistical analyses were conducted in R v. 3.6.0 (R Development Core Team 2020). To investigate the effect of predation risk on behavioral lateralization (i.e., how mean *L*_A_ varied among treatments after injection and its relationship with the pre-stimulus intensity), we applied beta general linear models (GLM), including post-stimulus *L*_A_ as response variable and predator treatment, pre- stimulus *L*_A_ and their interaction as predictors. We ran four chains with 4000 post burn-in samples, and we validated chain convergence visually. We used the default, non-informative priors set by the brm function in the *brms* R package (Bürkner 2017). Since beta GLM requires data within the range 0–1, we transformed both pre- and post-stimulus *L*_A_ indexes (Smithson and Verkuilen 2006; Douma and Weedon 2019).

The L_R_ index was assessed before stimulus injection, to explore the rotational lateralization at the population level, and after injection to investigate its potential variation among treatments. In both cases, we adopted a non-parametric approach using either one-sample Wilcoxon’s test (differences from zero) or Kruskal–Wallis’s rank sum tests (differences among treatments), respectively. *L*_A_ index before stimulus injection was also explored using Wilcoxon’s test.

To explore the effects of predation cues on the level of activity of tadpoles, we used generalized linear mixed models (GLMMs). The response variables were “total time frozen” and “average acceleration” after cue injection. Fixed factors included predator treatment (the type of cue injected, five levels) and the respective pre-stimulus response as covariate. The trial was included as a random effect. A gaussian distribution was adopted for “total time frozen” (LMM), while a gamma distribution was adopted for “average acceleration”; to improve the normality of residuals, in the latter case the covariate was not included in the final model. Confidence intervals, estimated means and planned comparisons with a control group (estimated differences) were obtained from fitted models using the R package *emmeans* (Lenth 2019). *T*- and *z*-ratios were used to compare estimated means (Lenth 2019).

Unless stated differently, data were reported as means ± standard errors.

## Results

Before being exposed to the stimuli, tadpoles’ mean *L*_R_ did not differ from zero (− 6.8 ± 5.5, *V* = 3432, *p* = 0.213), while the intensity of lateralization (*L*_A_) was significantly different (52.77 ± 2.84, *V* = 7875, *p* < 0.0001).

**Fig. 1:**
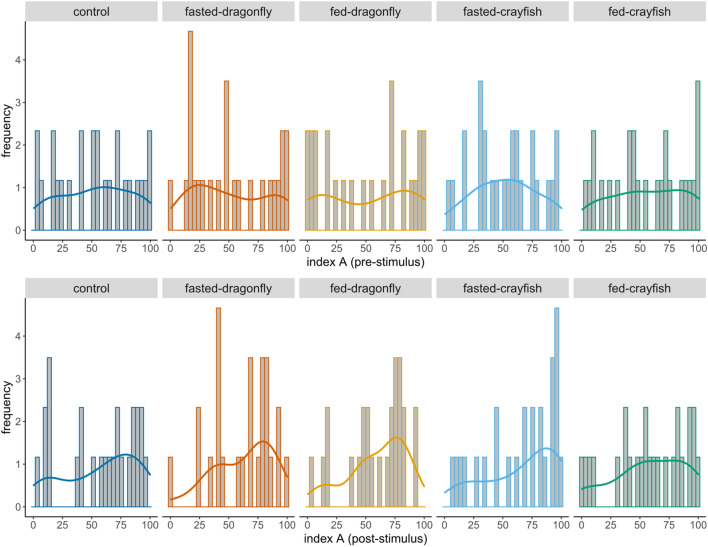
Distribution of both pre- (above) and post-stimulus (below) of the intensity of lateralization (*L*_A_ index), for all treatments (*n* = 125). Coloured lines represent smoothed density estimates (kernel density estimate) of data for each treatment

Neither post-stimulus mean *L*_R_ (*χ*^2^ = 4.829, df = 4, *p* value = 0.305) nor post-stimulus mean *L*_A_ (Table 1) differed among treatments. Nonetheless, smoothed density estimates showed a tendency of *L*_A_ to increase, with low (< 25) and high (> 75) scores becoming, respectively, less and more frequent in tadpoles exposed to the cues of both fed and fasted dragonfly larvae (Fig. 1).

**Table 1:** Post-stimulus mean *L*_A_ index and 95% highest mean posterior density intervals (HPDs) estimated from beta regression models for all treatments (*n* = 125)

Treatment	Estimated mean	Lower HPD	Upper HPD	Estimated odd ratio	Lower HPD (odd ratio)	Upper HPD (odd ratio)
Control	0.549	0.441	0.652			
Fasted-dragonfly	0.598	0.495	0.701	0.816	0.411	1.43
Fed-dragonfly	0.559	0.449	0.664	0.952	0.452	1.63
Fasted-crayfish	0.644	0.540	0.740	0.671	0.307	1.15
Fed-crayfish	0.566	0.459	0.668	0.935	0.441	2.07

Estimated comparisons with controls are reported as odd-ratios and their associated HPDs; a ratio close to 1 indicates a lack of difference between the treatment and control group. Since the unit is included in all intervals, the mean post *L*_A_ of each treatment does not differ from the control’s. Estimates are reported at the original scale (back transformed)

**Fig. 2:**
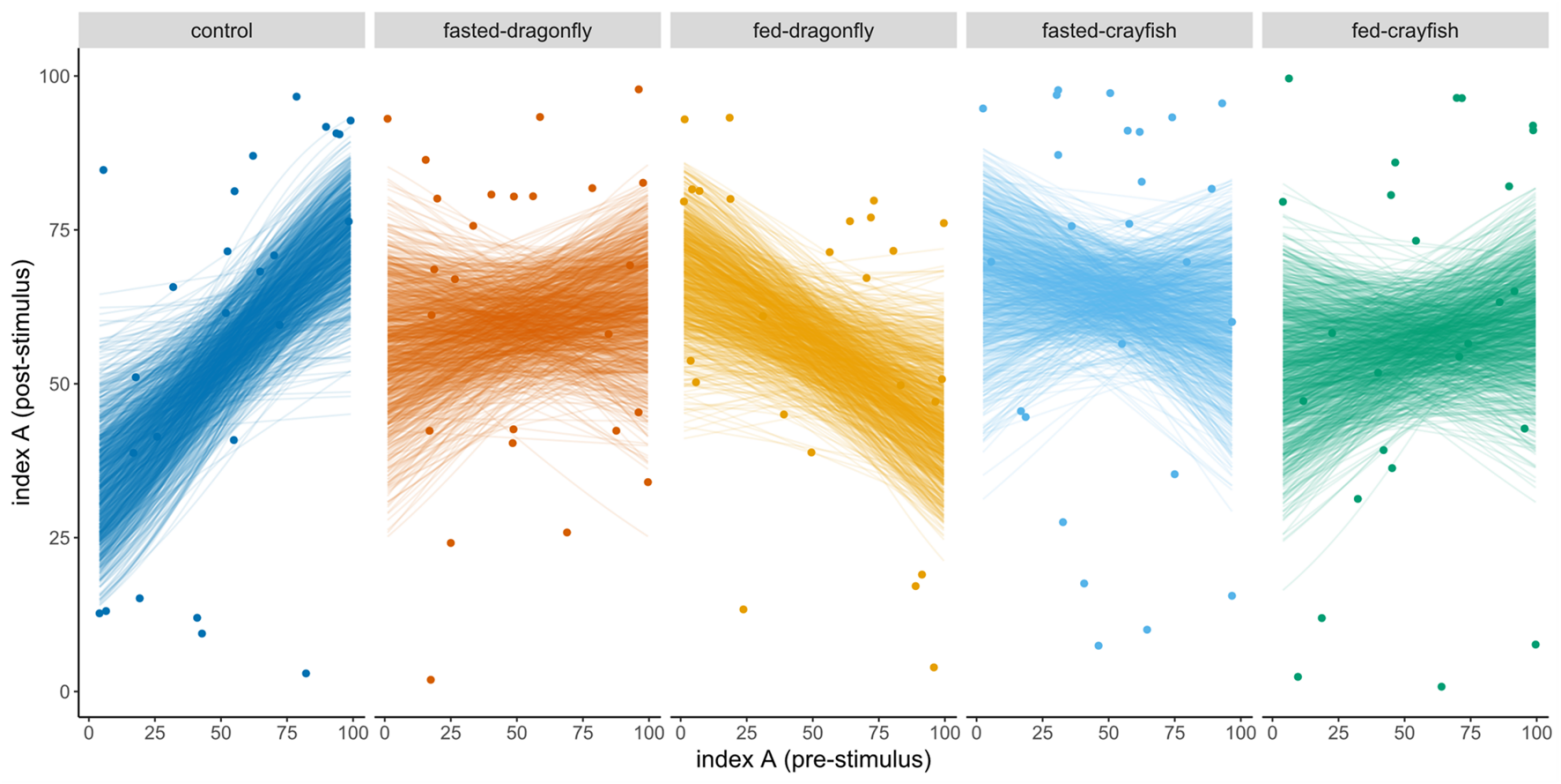
Estimated regression slopes from beta GLM with visualization of the uncertainty in the parameter estimates (one value is reported for each simulated regression, *n* = 1000, for each treatment)

The relationship between post and pre-stimulus *L*_A_ differed among treatments: the slope of the fed-dragonfly and, to a much lesser extent, fasted-crayfish treatments showed opposite pattern respect to the control group, indicating the decrease of post-stimulus *L*_A_ for increasing values of the pre-stimulus index (Table 2, Fig. 2). All the other treatments did not show a significantly different slope when compared to controls (Table 2, Fig. 2).

**Table 2:** Post stimulus mean regression slopes and 95% highest posterior density intervals (HPDs) for *L*_A_ index, estimated from the beta regression model, as a function of both pre-stimulus *L*_A_ and treatment (interaction in the model)

Signal	Contrast	Pre-stimulus *L*_A_ (slope)	Lower HPD	Upper HPD
Control		1.830	**0.437**	**3.242**
Fasted-dragonfly		0.297	− 1.106	1.651
Fed-dragonfly		− 1.025	− 2.266	0.107
Fasted-crayfish		− 0.366	− 2.003	1.202
Fed-crayfish		0.335	− 1.058	1.687
	Control—(fasted-dragonfly)	1.518	− 0.479	3.428
	Control—(fed-dragonfly)	2.865	**1.1019**	**4.750**
	Control—(fasted-crayfish)	2.189	**0.0805**	**4.335**
	Control—(fed-crayfish)	1.499	− 0.448	3.488

Estimated contrasts with control are reported as differences in slope and relative HPDs (*n* = 125). Values in bold indicate significant differences from zero or from the slope of the control group

**Fig. 3:**
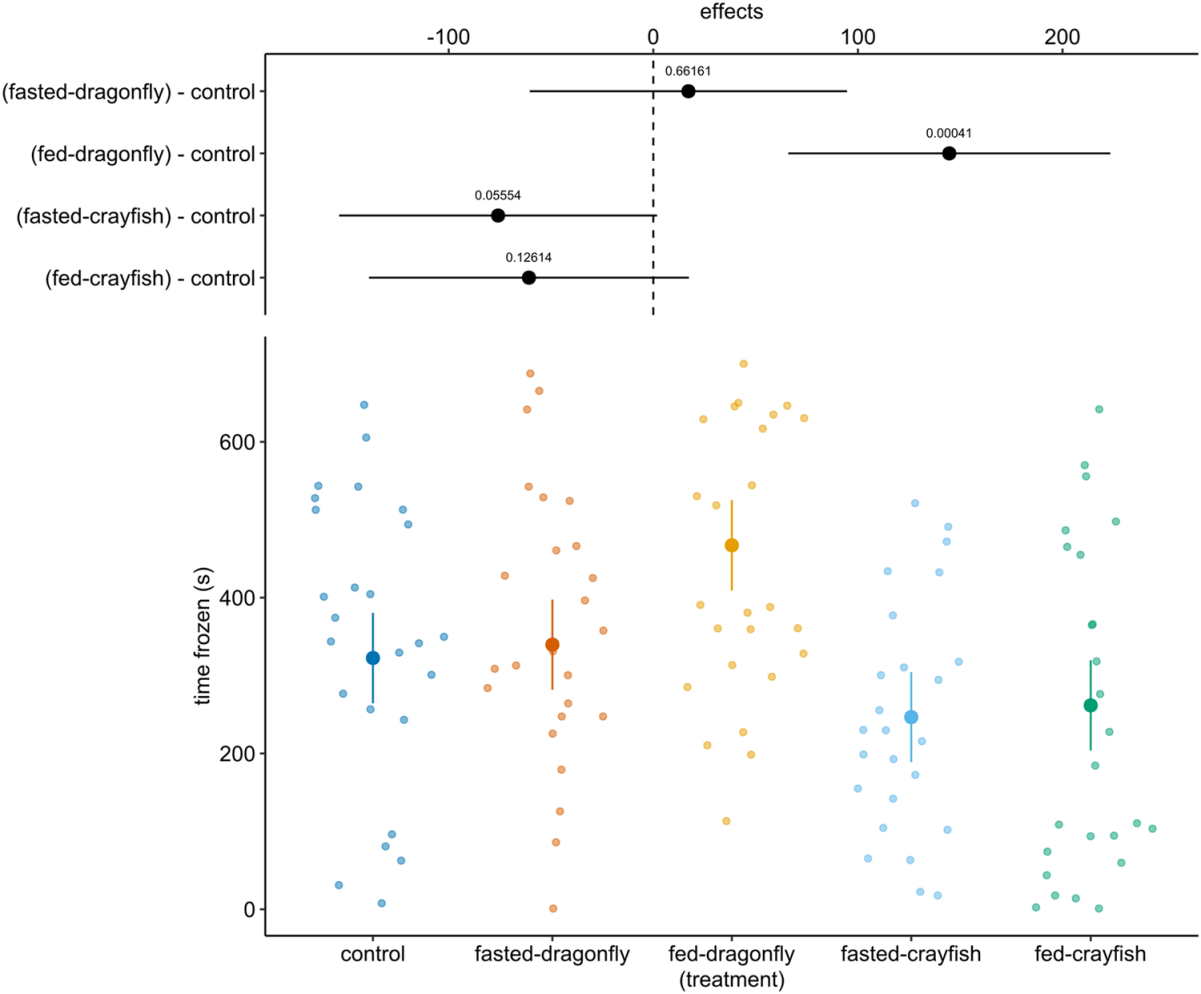
Estimated means and 95% confidence intervals (large points and coloured lines) for time frozen as a response variable in LMM. Above are reported the estimated means and 95% confidence intervals for comparison, as a difference from control, with each treatment; the values reported were obtained from the model using *emmeans* package (*n* = 25 per treatment)

The model revealed a significant effect of treatment (*χ*^2^ = 39.79, df = 4, *p* < 0.0001) and pre-stimulus activity (*χ*^2^ = 72.90, df = 1, *p* < 0.0001; slope = 0.56 ± 0.06, df = 119, *t* = 8.53, *p* < 0.0001) on “total time frozen” after stimulus injection. Fed-dragonfly larvae induced the sharpest increase respect to controls (estimated difference = 144.7 ± 39.7, *t*-ratio = 3.64, *p* = 0.0004: Fig. 3); on the opposite both fasted and fed-crayfish induced a reduction of the time spent motionless (− 76 ± 39.2, *t*-ratio = − 1.95, *p* = 0.05 and − 60.8 ± 39.4, *t*-ratio = − 1.54, *p* = 0.12 respectively, Fig. 3). The fasted-dragonfly treatment did not differ from the control group (estimated difference = 17.2 ± 39.1, *t*-ratio = 0.49, *p* = 0.66).

**Fig. 4:**
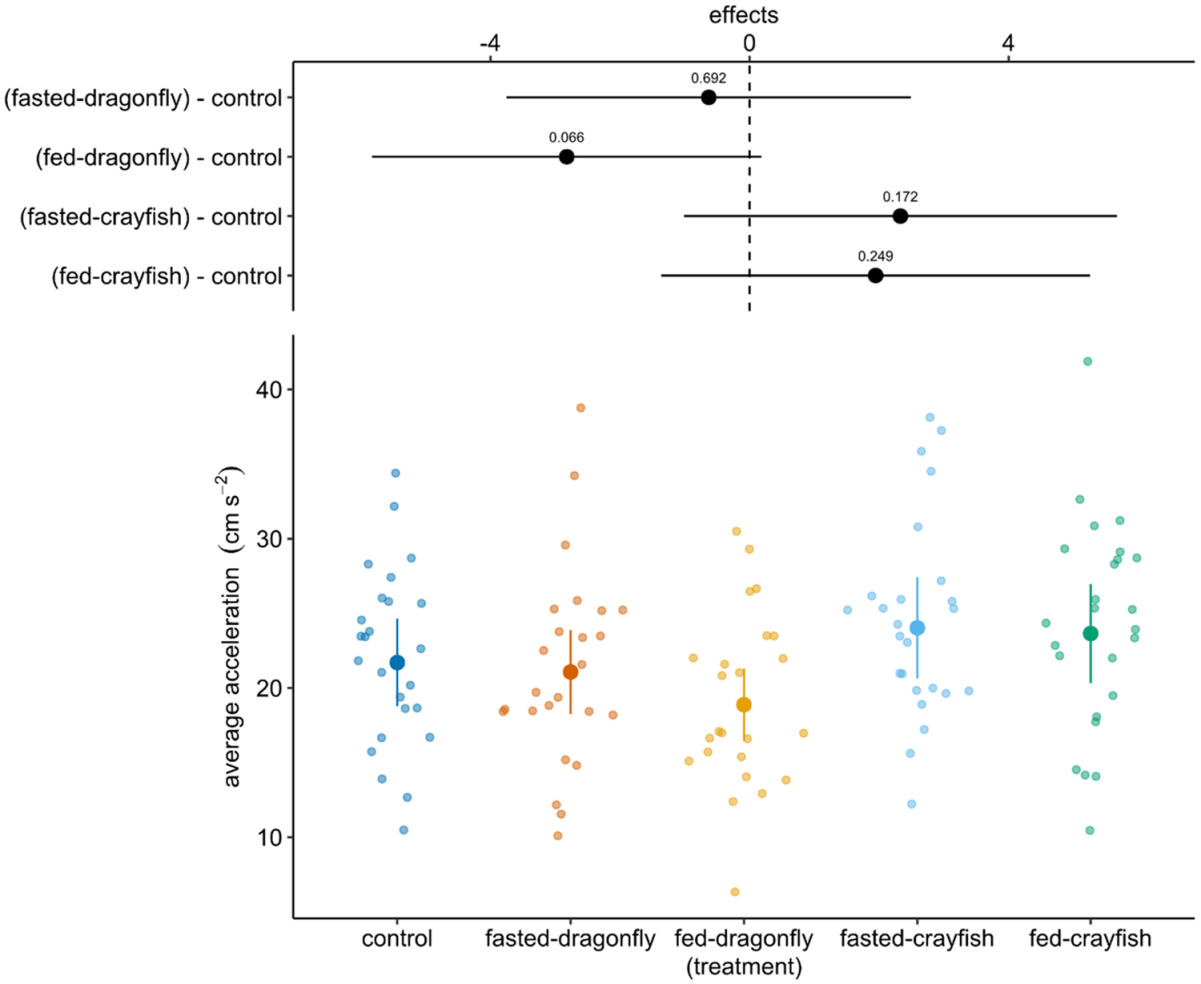
Estimated means and 95% confidence intervals (points and coloured lines) for average acceleration as a response variable in GLMM. Above are reported the estimated means and 95% confidence intervals for comparison, as a difference from control, with each treatment; the values reported were obtained from the model using *emmeans* package (*n* = 25 per treatment)

Treatments also affected the average post-stimulus acceleration (*χ*^2^ = 13.05, df = 4, *p* = 0.011). Exposure to fed-dragonfly larvae slightly lowered tadpoles’ average acceleration in comparison to controls (estimated difference = − 2.82 ± 1.59, *z*-ratio =− 1.84, *p* = 0.065), while all other treatments did not show sensible differences with respect to control group (fasted-dragonfly – control = − 0.63 ± 1.53, *z*-ratio = − 0.396, *p* = 0.692; fasted-crayfish – control = 2.33 ± 1.71, *z*-ratio = 1.36, *p* = 0.172; fed-crayfish – control = 1.956 ± 1.69, *z*-ratio = 1.15, *p* = 0.249; Fig. 4).

## Discussion

When facing an attack, prey usually respond using two main defensive behaviours: *freezing*, i.e., a sudden arrest of movement, and *fleeing* in the opposite direction, or a combination of the two (Edut and Eilam 2004). The most frequently reported behavioural response of tadpoles, as assessed by exposing them to predator and prey-borne chemical signals (Hettyey et al. 2015), is a reduction in activity levels (e.g.: Van Buskirk 2001; Steiner 2007; Gazzola et al. 2017, 2018b). However, the analysis of individual trajectories indicates that, alternatively, tadpoles may also incorporate protean elements into their movement (Gazzola et al. 2021; Castellano et al. 2022), i.e., change frequently the direction of swimming to prevent predators from anticipating their position and lower their targeting accuracy (Jones et al. 2011). Pre-existing turning biases may affect both predator detection and defensive performances (Cantalupo et al. 1995; Lippolis et al. 2002; Rogers et al. 2013). Despite population-level biases have been recorded for several gregarious species (Vallortigara et al. 1999; Ghirlanda and Vallortigara 2004), in our study Balearic green toad tadpoles did not show a bias in a predominant direction, although lateralization occurred at the individual level, as shown by the intensity index (*L*_A_).

Perceived predation risk was the highest in tadpoles exposed to the combined olfactory cues of attacked conspecifics and native predators (fed dragonfly larvae), which elicited both changes in the intensity of lateralization and a marked reduction in tadpoles’ activity level. While the latter response was expected, having previously been recorded by several authors and being widespread in the tadpoles of sympatric anuran species (common water frog *Pelophylax* kl. *esculentus*, Gazzola et al. 2018a; agile frog *Rana dalmatina,* Gazzola et al. 2018b; Italian agile frog *Rana latastei*, Scribano et al. 2020), changes in individual lateralization were less straightforward and may imply defensive strategies more complex than previously reported.

Although appreciable only qualitatively (using kernel density estimates), the increase in lateralization intensity was consistent with previous studies on fish (Brown et al. 2011), suggesting that tadpoles may tend to keep the predator on a specific eye side, possibly that preferred for processing information related to potential threats (Ferrari et al. 2015a). Notwithstanding this general trend, the inverse relationship between pre- and post-stimulus lateralization intensities pointed out that high predation pressure urged individual tadpoles to change their fleeing behaviour in a subtler way, adopting a swimming pattern different from or opposite to that shown in the pre-stimulus phase, namely zigzagging vs. moving mainly straight up and vice versa.

Predator–prey interactions imply the mutual exchange of predator-borne disturbing signals and attack-provoking cues unintentionally emitted by prey (Dixon 1998). Freezing and, whenever possible, concealment, are intended to reduce or nullify the emission of provoking cues, while protean behaviours, i.e., rapid erratic movements (Chance and Russel 1959), should disturb the reception of prey-borne cues by the predator and make the direction of movement of prey unpredictable (Dixon 1998). Our results suggest that these alternative (or combined) responses describe only partially the complexity of defensive behaviour, which may include the display of movement patterns different from or opposite to those potentially already recorded by predators lying in ambush (i.e., the emission of contradictory attack-evoking signals subsequent to the identification of the predator).

While frogs’ responses to alien crayfish have been widely investigated (Gomez-Mestre and Diaz-Paniagua 2011; Nunes et al. 2013; Gazzola et al. 2021), to our knowledge nothing is known about the capability of green toads of responding to non-native predators. Increased activity in tadpoles exposed to both fasted and fed-crayfish indicates that these predators were not detected effectively. Since an activity increase is often observed in fasting tadpoles, which are more pressed by foraging needs (Horat and Semlitsch 1994; Fraker 2008), as recorded for fasted *Rana latastei* tadpoles exposed to the cue of red swamp crayfish (personal observations), green toad tadpoles might have perceived crayfish cue as a food odour stimulus.

Game-theoretical models suggest that, at the population level, the occurrence of 10–35% of individuals showing the minority bias is favored by selective pressures favouring unpredictable defensive responses (Ghirlanda and Vallortigara 2004). More recently, fish lateralization has been demonstrated to vary according to predation risk or environmental conditions. High-risk conditions, simulated by exposing juvenile fish to injured conspecific signals for 4–9 days, were shown to increase the intensity of lateralization (Ferrari et al. 2015b, 2017; Chivers et al. 2016), although the direction and within-treatment consistency of the bias can differ (Chivers et al. 2016). The intensity of light to which pregnant females are exposed close to parturition affects the lateralization of new-born goldbelly topminnow *Girardinus falcatus* (Dadda and Bisazza 2012), while, using edible frogs *Pelophylax esculentus*, Lucon-Xiccato et al. (2020) have also recently shown that vegetation cover during the embryonic stage can affect both the directionality and intensity of tadpole lateralization.

Environmental stressors, such as high carbon dioxide concentration (Nilsson et al. 2012) or hypoxic conditions (Lucon-Xiccato et al. 2014) can alter population-level lateralization. Also a brief (1.5 h) period of monocularly has been reported to invert the magnetic compass orientation in European robin *Erithacus rubecula* (Gehring et al. 2017).

All these studies suggest that, as with other behavioural traits (DeWitt and Scheiner 2004), lateralization may be more flexible than previously believed, showing some variation in response to the environmental conditions to which individuals are exposed during development. Our results suggest that contextual predation threat may induce very rapid changes in the expression of asymmetries at the individual level, as part of the complex defensive strategies adopted by prey in the attempt to escape predators. Further studies on a moment-to-moment basis are needed to verify whether other environmental pressures can elicit immediate changes in the expression of behavioural asymmetries.
